# Mild and Moderate Traumatic Brain Injury and Repeated Stress Affect Corticosterone in the Rat

**DOI:** 10.1089/neur.2020.0019

**Published:** 2020-10-21

**Authors:** Rachel K. Rowe, J. Bryce Ortiz, Theresa Currier Thomas

**Affiliations:** ^1^ Barrow Neurological Institute, Phoenix Children's Hospital, Phoenix, Arizona, USA.; ^2^ Department of Child Health, University of Arizona College of Medicine Phoenix, Phoenix, Arizona, USA.; ^3^ Phoenix Veteran Affairs Health Care System, Phoenix, Arizona, USA.

**Keywords:** chronic, concussion, corticosterone, mild, moderate, stress, traumatic brain injury

## Abstract

Traumatic brain injury (TBI) survivors suffer from a range of morbidities, including post-traumatic endocrinopathies that can cause physical and mental changes in patients, greatly compromising quality of life. This study tested the hypothesis that mild and moderate diffuse TBI leads to chronic deficiencies in corticosterone (CORT) regulation following repeated exposure to restraint stress over time. Young adult male rats (*n* = 9–11/group) were subjected to mild or moderate TBI induced by midline fluid percussion injury (mFPI) or control sham surgery. At 6 and 24 h post-injury, both mild and moderate TBI resulted in elevated resting plasma CORT levels compared with uninjured shams. Independent of TBI severity, all rats had lower resting plasma CORT levels at 7, 14, 28, and 54 days post-injury compared with pre-surgery baseline CORT. Circulating levels of CORT were also evaluated under restraint stress and in response to dexamethasone (DEX), a synthetic glucocorticoid. Independent of TBI severity, restraint stress elevated CORT at 30, 60, and 90 min post-stressor initiation at all post-injury time-points. A blunted CORT response to restraint stress was observed with lower CORT levels after restraint at 28 and 54 days compared with 7 days post-injury (DPI), indicative of habituation to the stressor. A high dose of DEX lowered CORT levels at 90 min post-restraint stress initiation compared with low-dose DEX, independent of TBI severity. These results support TBI-induced CORT dysregulation at acute time-points, but additional studies that investigate the onset and progression of endocrinopathies, controlling for habituation to repeated restraint stress, are needed to inform the diagnosis and treatment of such morbidities in TBI survivors.

## Introduction

Traumatic brain injury (TBI) is initiated by a mechanical injury followed by ensuing cellular cascades that lead to the development of acute deficits and chronic morbidities.^1,2^ TBI survivors suffer from a range of morbidities that include post-traumatic endocrinopathies.^3^ Although endocrinopathies were once thought to be rare, they are now reported over a wide range, with 2–90% of TBI survivors reporting endocrine dysregulation.^3–5^ Endocrinopathies can cause impairments that can substantially compromise quality of life in TBI survivors.^3,6,7^ The risk factors and pathophysiology of TBI-induced endocrinopathies are not yet fully understood, which limits the development and refinement of therapeutic targets and rehabilitative strategies. Pre-clinical studies that investigate the onset and progression of these endocrinopathies may provide translational insights that inform the treatment of such morbidities in TBI survivors.

Hypothalamic-pituitary-adrenal (HPA) axis dysregulation has been observed in clinical and pre-clinical studies as a result of TBI.^4,8–12^ Hypopituitarism, defined as decreased production, release, circulation, and/or regulation of one or more hormones produced by the pituitary, results in endocrine dysfunction in TBI survivors.^13,14^ Although previously associated with moderate to severe TBI, clinical data support similar rates of hypopituitarism following mild TBI.^15,16^

TBI is not an isolated injury but a lifelong event, and survivors, including soldiers, athletes, and victims of domestic violence, are often exposed to repeated stressors after injury. Exposure to a stressor leads to physiological and behavioral responses that aim to aid in adaptation to the stressor.^17,18^ One of these responses involves the release of glucocorticoids, a class of steroid hormones that lead to changes in the brain and body.^19,20^ The main glucocorticoid in rodents is corticosterone (CORT). Because of their pleiotropic effects, glucocorticoids are rigorously regulated, in part, through negative feedback responses on the HPA axis. Impaired negative feedback can lead to a prolonged release of CORT following a stressful experience.^21–23^

Daily repeated homotypic restraint stress has historically been implemented in rodents, leading to habituation and an attenuation of the stress response (e.g., circulating CORT) over time.^24–26^ Data suggest homotypic restraint stress administered intermittently (e.g., 5 days with a 2-day rest) produces a more robust CORT response after the rest than daily restraint stress, thus decreasing habituation/attenuation of the CORT response.^27,28^ Therefore, we implemented intermittent homotypic restraint stress with a minimum of a 1-week rest period in attempt to avoid habituation/attenuation of the CORT response. We previously reported that diffuse TBI leads to endocrine dysregulation in rats at 2 months post-TBI.^12^ The objective of the current study was to extend these findings and test the hypothesis that mild and moderate diffuse TBI lead to chronic dysregulation of CORT and that repeated sampling would identify the onset of CORT dysregulation.

## Methods

### Study design and rigor

Studies were conducted in accordance with guidelines established by a University of Arizona Institutional Animal Care and Use Committee (IACUC) and National Institutes of Health (NIH) guidelines for the care and use of laboratory animals. Studies are reported following the ARRIVE (Animal Research: Reporting of In Vivo Experiments) guidelines. Randomization was achieved by assigning animals to treatment groups before the initiation of the study. Data collection stopped at predetermined final end-points based on days post-injury (DPI). CORT calculations were made by investigators blinded to treatment groups.

### Animals

Young adult male (10–12 weeks old; 350–370 g) Sprague-Dawley rats (Harlan Laboratories, Inc., Indianapolis, IN, USA) were used (*n* = 34). Rats were pair-housed in a 12-h light/12-h dark cycle (06:00/18:00) at a constant temperature (23°C ± 2°C) with food and water available *ad libitum*. Rats were acclimated following shipment for at least 1 week. After surgery, rats were evaluated daily for post-operative care by a physical examination and documentation of each animal's condition. Procedures were approved by the University of Arizona IACUC (protocol 18-384).

### Midline fluid percussion injury (mFPI)

Rats were subjected to midline fluid percussion injury (mFPI) as previously described.^29–34^ This injury model was selected because mFPI reproduces diffuse axonal injury without cavitation or gross histopathology, a hallmark of clinical diffuse TBI.^35^ Rats were anesthetized using 5% isoflurane in 100% oxygen for 5 min then placed in a stereotaxic frame with continuous isoflurane at 2.0% via nose cone. Body temperature was maintained using a Deltaphase^®^ isothermal heating pad (Braintree Scientific, Inc., Braintree, MA, USA). A midline incision was made, and fascia was removed from the skull. A trephine (4.8 mm outer diameter) was used for the craniectomy, centered on the sagittal suture between bregma and lambda without disruption of the dura. An injury hub prepared from the female portion of a Luer-Loc needle hub was fixed over the craniectomy using cyanoacrylate gel and methyl-methacrylate (Hygenic Corp., Akron, OH, USA). Rats were then placed in a heated recovery cage and monitored until ambulatory.

For injury induction, rats were re-anesthetized (60–90 min after surgery) with 5% isoflurane delivered for 3 min. The dura was visually inspected to make sure it was intact with no debris. The hub was filled with normal saline and attached to the male end of the fluid percussion device (Custom Design and Fabrication, Virginia Commonwealth University, Richmond, VA, USA). After the return of a pedal withdrawal response, an injury averaging 2.2 atm for a moderate TBI injury (Mod TBI) or 1.2 atm for a mild TBI injury (Mild TBI) was administered by releasing the pendulum onto the fluid-filled cylinder.^12,35^ Shams underwent the same surgical procedures except the pendulum was not released after a positive pedal withdrawal response. Rats were monitored for the presence of a forearm fencing response, and righting reflex times were recorded for the injured rats as indicators of injury severity.^36^ The righting reflex time is the total time from the initial impact until the rat spontaneously rights itself from a supine position.

After righting, brains were inspected for herniation, hematomas, and dura integrity. Inclusion criteria included uniform herniation with no dura breach. The incision was cleaned using saline and closed using staples. Inclusion criteria for Mod TBI rats: positive fencing response and righting reflex time between 6 and 10 min (528.4 ± 159 sec).^12^ Inclusion criteria for Mild TBI rats: righting reflex time greater than 2 min but less than 6 min (249.8 ± 56.3 sec).^37^ Sham rats recovered a righting reflex immediately. One rat was excluded as a technical failure for unilateral herniation and dura breach. One rat died within 5 min of a moderate TBI, one rat died within 48 h of a moderate TBI, and one rat had excessive bleeding from the sagittal sinus immediately following a mild TBI and was euthanized; all four were excluded from the study. Final group numbers were sham *n* = 9, Mild TBI *n* = 11, and Mod TBI *n* = 10.

### Post-operative weights

Weights were recorded on the day of surgery. Rats were monitored for 3 DPI with a physical examination, thorough examination of the wound site, and evaluation of changes in post-operative weight. Terminal weights were recorded at 58 DPI.

### Restraint stress and blood collection

Rats were exposed to restraint stress and blood draws at predetermined post-injury time-points. For the study design see Supplementary Figure S1. The day of surgery (between 04:00 and 07:00), rats were anesthetized for 5 min with isoflurane, secured in a stereotaxic frame, and a pre-surgery (baseline) blood sample was collected (250 μL) from the lateral tail vein. At 6 h (14:00–15:30) and 24 h post-injury (07:00–09:00), rats were loosely secured in a restrainer (<5 min) and blood was collected from the lateral tail vein (250 μL). At 7, 14, 28, 54, and 56 DPI, rats underwent restraint stress and blood collection. Samples were collected immediately following light onset (restraint was initiated between 07:30 and 09:30, blood was collected between 7:30 and 12:00) to control for CORT having diurnal peaks.^38^

Briefly, a rat was transported in its home cage to a procedure room and placed tightly into a flat-bottom Plexiglas restraining tube (Head Access Rodent Restrainer, Stoelting, Wood Dale, IL, USA). The process was strictly followed at all time-points and took approximately 5 min from when the animal was disturbed until the first blood draw was acquired. Rats remained in the restrainer for a total of 90 min (30 min of tight restraint, followed by 60 min of loosened restraint) and blood was repeatedly collected (250 μL) at 30, 60, and 90 min.^12^ Blood samples were collected into tubes coated with ethylenediaminetetraacetic acid (EDTA), centrifuged at 3000 revolutions per minute (rpm) for 10 min at 4°C, divided into three labeled tubes, and stored at −20°C.

### Dexamethasone treatment

At 56 DPI, rats were randomly assigned to a low (0.01 mg/kg; sham *n =* 5, Mild TBI *n =* 5, Mod TBI *n =* 5) or high (0.1 mg/kg; sham *n =* 4, Mild TBI *n =* 6, Mod TBI *n =* 5) dose of dexamethasone (DEX; Dexamethasone Injectable sc-362917Rx, Santa Cruz Biotechnology, Sana Cruz, CA, USA). DEX was administered subcutaneously (06:00) to investigate effectiveness of HPA axis negative feedback mechanisms.^39^ This dose was guided by a literature review of rats receiving DEX prior to or following experimental TBI^40–43^ and our previously published work.^12^ Two hours post-injection the restraint stress paradigm was repeated.

### Corticosterone

Plasma CORT levels were quantified using enzyme-linked immunosorbent assay (ELISA) kits purchased through EnzoLife Sciences, Inc. (Farmingdale, NY, USA). CORT samples were run in triplicate following the manufacturer's instructions. Plasma samples were diluted 1:50 per manufacturer's recommendations for the CORT ELISA. Samples with the DEX treatment were run at higher concentrations to reach detectable limits of the ELISA (experiments were repeated at dilutions of 1:10–1:25).

### Statistical analysis

Data were analyzed using GraphPad-Prism version 8.0.1. Differences in righting reflex times, weights, and baseline CORT were measured using a one-way analysis of variance (ANOVA) followed by Tukey's multiple comparison test when appropriate. Mixed-effects models, followed by Tukey's post hoc analysis, when appropriate, were used for CORT because levels were measured at multiple time-points from the same animals. We tested two categorical variables and their interaction as fixed effects (injury, time, injury × time), with subject (individual rat) as the random effect in each model to account for collinearity between repeated measurements on the same individuals. Chi-square tests were used to compare the fit of mixed-effects models to models with identical fixed effects but that ignored the within-subject correlation between repeated measurements. CORT levels after DEX and the change in CORT after DEX compared with CORT at 54 DPI were analyzed with a two-way ANOVA followed by Tukey's multiple comparisons. Data sets were screened using the extreme studentized deviate method to identify potential outliers. Only outliers from measurement or technical error in blood collection or ELISA preparation were removed from analyses and reported in the Results section; outliers that could not be attributed to measurement or technical error were appropriately retained in the analyses. Statistical significance was assigned when *p* < 0.05.

## Results

### Diffuse TBI suppressed acute neurological reflexes but did not alter chronic weight gain

**FIG. 1.**
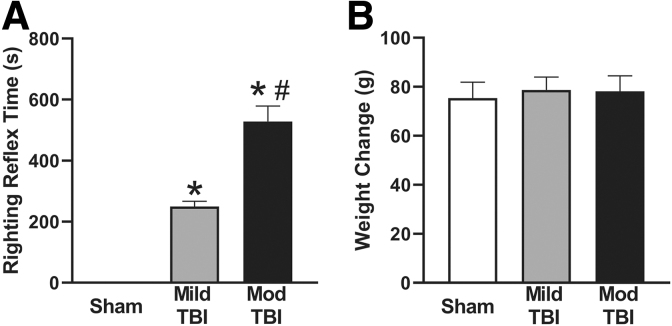
Diffuse TBI suppressed acute neurological reflexes but did not alter chronic weight gain. **(A)** Righting reflex times were measured following midline fluid percussion injury. Both TBI groups had longer righting reflex times than uninjured shams. Mod TBI resulted in longer righting reflex times compared with rats subjected to Mild TBI. (* denotes significance compared with sham, # denotes significant compared with Mild TBI). **(B)** There were no differences in weight gain measured as the change in weight from pre-surgery to terminal time-points. All data are presented as mean ± SEM, *p* < 0.05. Mild TBI, mild TBI injury; Mod TBI, moderate TBI injury; SEM, standard error of the mean; TBI, traumatic brain injury.

There was an injury effect on righting reflex time (F[2,27] = 69.14, *p* < 0.0001; Fig. 1A), with both Mild TBI and Mod TBI rats having longer times than uninjured shams, and Mod TBI rats having times an average of 278.6 sec longer than those of Mild TBI rats. There were no differences in weight change among groups (F[2,27] = 0.086, *p =* 0.918; Fig. 1B).

### Diffuse TBI resulted in elevated acute CORT levels independent of stress

**FIG. 2.**
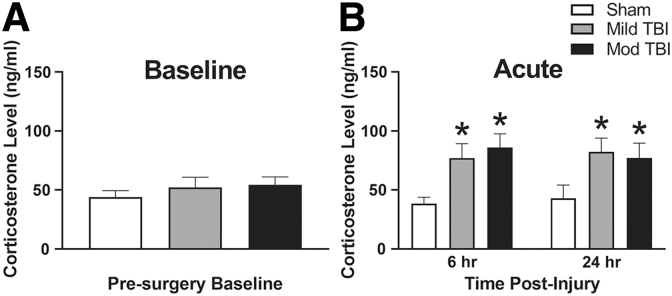
Diffuse TBI resulted in increased acute CORT levels independent of stress. **(A)** There were no differences in CORT among groups measured at pre-surgery baseline. **(B)** Both Mild TBI and Mod TBI resulted in higher levels of CORT at 6 h and 24 h post-injury compared with uninjured shams. All data are presented as mean ± SEM, *p* < 0.05. (* denotes significance compared with sham). CORT, corticosterone; Mild TBI, mild TBI injury; Mod TBI, moderate TBI injury; SEM, standard error of the mean; TBI, traumatic brain injury.

CORT was measured: 1) at pre-surgery baseline; 2) acutely at 6 and 24 h post-injury; and 3) chronically at 7, 14, 28, and 54 DPI. There were no differences in pre-surgery CORT levels among groups (F[2,27] = 0.545, *p =* 0.586; Fig. 2A). After accounting for the within-subject correlation between repeated measures on the same individuals via random effects ($\chi_{1}^{2}=7.706$, *p =* 0.006), an injury effect on acute CORT levels was supported at 6 and 24 h post-injury (F[2,25] = 5.138, *p =* 0.014; Fig. 2B); TBI groups had on average 39.8 ng/mL higher CORT levels compared with shams, regardless of injury severity. A time effect on acute CORT levels was not supported (F[1,24] = 0.002, *p =* 0.966; Fig. 2B), nor was an interaction between injury and time (F[2, 24] = 0.430, *p =* 0.656; Fig. 2B) collectively, indicating that TBI-induced CORT remained elevated across acute time-points. Outliers not included in analyses: sham 6 h, *n =* 1; sham 24 h, *n =* 1; Mild TBI 6 h, *n =* 1; Mod TBI 6 h, *n =* 1; Mod TBI 24 h, *n =* 1.

### Chronic CORT levels were decreased as a function of stress, but not injury

**FIG. 3.**
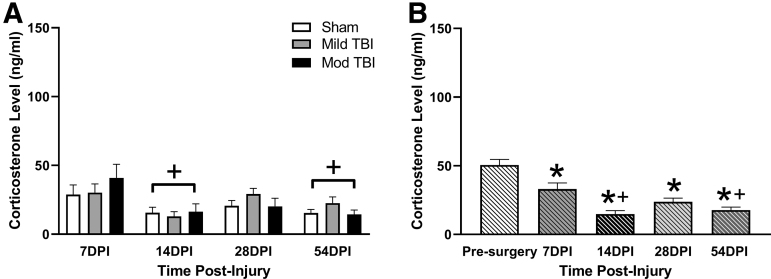
Chronic CORT levels were decreased as function of stress, but not injury. **(A)** TBI did not lead to changes in CORT levels measured pre-stress (time 0). There was a significant time effect and CORT levels at 14 DPI and 54 DPI were lower than CORT at 7DPI. (* denotes significance compared with 7 DPI) **(B)**. We analyzed pre-stress CORT (0 min) independent of TBI compared with pre-surgery CORT and found all chronic time-points (7, 14, 28, and 54 DPI) had lower levels of CORT compared with CORT levels measured at the pre-surgery baseline. CORT at 14 DPI and 54 DPI was also lower when compared with CORT levels at 7 DPI. (* denotes significance compared with sham, + denotes significant compared with 7 DPI). All data are presented as mean ± SEM, *p* < 0.05. CORT, corticosterone; DPI, days post-injury; Mild TBI, mild TBI injury; Mod TBI, moderate TBI injury; SEM, standard error of the mean; TBI, traumatic brain injury.

There was no injury effect on chronic CORT levels (F[2,27] = 0.383, *p =* 0.686; Fig. 3A). There was a time effect on chronic CORT (F[3,75] = 7.590, *p =* 0.0002; Fig. 3A) that was on average18.3 ng/mL lower at 14 DPI and 15.33 ng/mL lower at 54 DPI compared with 7 DPI for all rats. There was no evidence that within-subject repeated measures were correlated ($\chi_{1}^{2}=0.960$, *p =* 0.327; Fig. 3A), and no interaction between injury and time (F[6,75] = 1.027, *p =* 0.415; Fig. 3A). We further analyzed CORT levels by combining treatment groups and evaluating CORT, independent of TBI, measured at time 0 (pre-stress) at 7, 14, 28, and 54 DPI. We compared these values with the pre-surgery baseline CORT levels to investigate if repeated exposure to the restraint stress paradigm resulted in lower chronic resting CORT independent of mild or moderate TBI. An overall effect on chronic CORT across time was supported (F[4,139] = 3.764, *p =* 0.0061; Fig. 3B). CORT levels at 7, 14, and 28 days were lower than pre-surgery levels and CORT levels at 14 days and 54 days were lower than both pre-surgery and 7-day levels, indicating a decrease in CORT over time regardless of treatment. Outliers not included in analyses: 7 DPI, *n =* 4; 14 DPI, *n =* 2.

### Restraint stress elevated CORT levels independent of diffuse TBI

**FIG. 4.**
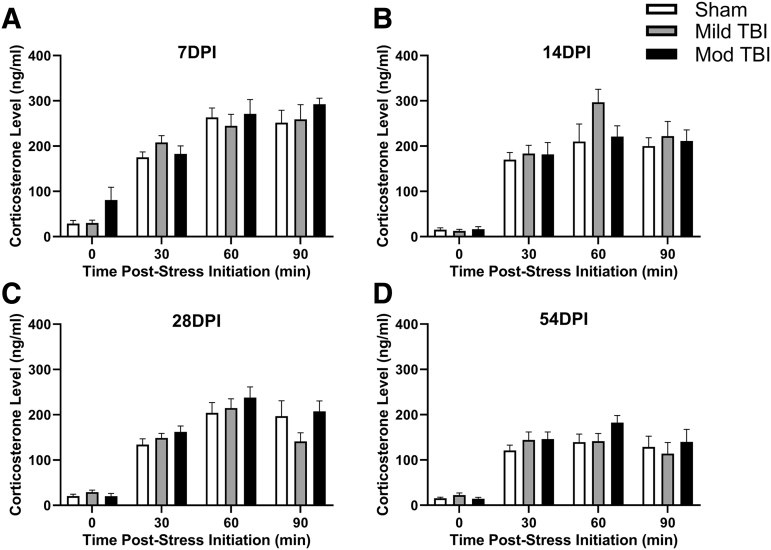
Restraint stress increased CORT levels independent of diffuse TBI. Rats were exposed to a restraint stress paradigm and blood was collected pre-stress (0 min) and at 30, 60, and 90 min post-stress. Regardless of TBI, exposure to the restraint stress paradigm increased CORT levels compared with pre-stress levels (0 min) at **(A)** 7 DPI, **(B)** 14 DPI, **(C)** 28 DPI, and **(D)** 54 DPI. All data are presented as mean ± SEM, *p* < 0.05. CORT, corticosterone; DPI, days post-injury; Mild TBI, mild TBI injury; Mod TBI, moderate TBI injury; SEM, standard error of the mean; TBI, traumatic brain injury.

CORT was measured immediately after placement in a restrainer (time 0) and 30, 60, and 90 min post-restraint stress initiation.^12^ This stress paradigm was utilized at 7, 14, 28, and 54 DPI. After accounting for the within-subject correlation between repeated measures via random effects at 7 DPI ($\chi_{1}^{2}=4.869$, *p =* 0.02), 14 DPI ($\chi_{1}^{2}=11.94$, *p =* 0.0005), 28 DPI ($\chi_{1}^{2}=15.92$, *p* < 0.0001), and 54 DPI ($\chi_{1}^{2}=13.98$, *p =* 0.0002), support existed for restraint stress leading to elevated CORT levels measured by a significant time effect on CORT in all rats at 7 DPI (F[3,76] = 78.43 *p* < 0.0001; Fig. 4A), 14 DPI (F[3,78] + 82.75, *p* < 0.0001; Fig. 4B), 28 DPI (F[3,78] = 106.0, *p* < 0.0001; Fig. 4C), and 54 DPI (F[3,79] = 59.53, *p* < 0.0001; Fig. 4D). There were no significant injury-dependent differences in CORT at 7 DPI (F[2,27] = 0.919, *p =* 0.411; Fig. 4A), 14 DPI (F[2,27] = 0.894, *p =* 0.421; Fig. 4B), 28 DPI (F[2,27] = 0.994, *p =* 0.383; Fig. 4C), or 54 DPI (F[2,27] = 0.595, *p =* 0.560; Fig. 4D). No support existed for an interaction between injury and time at 7 DPI (F[6,76] = 0.896, *p =* 0.503; Fig. 4A), 14 DPI (F[6,78] = 1.264, *p =* 0.284; Fig. 4B), 28 DPI (F[6, 78] = 1.903, *p =* 0.091; Fig. 4C), or 54 DPI (F[6,79] = 0.817, *p =* 0.560; Fig. 4D). Outliers not included in analyses: sham 0 min, *n =* 1; Mild TBI 0 min, *n =* 1; Mild TBI 60 min, *n =* 1; 90 min, *n =* 1; Mod TBI 90 min, *n =* 1.

### Rats habituated to repeated restraint stress independent of TBI

**FIG. 5.**
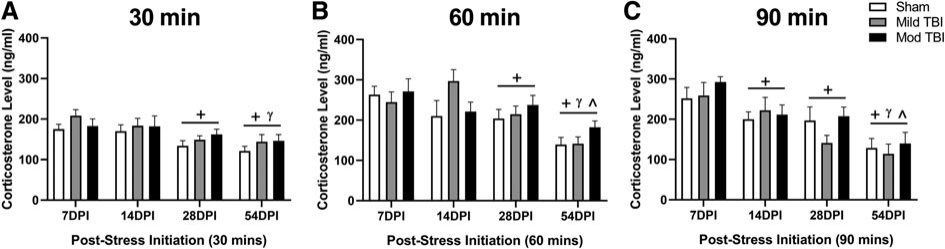
Rats habituated to repeated restraint stress independent of TBI. **(A)** Following 30 min of restraint stress, CORT levels were lower at 28 DPI compared with 7 DPI and lower at 54 DPI compared with both 7 and 14 DPI. **(B)** Following 60 min of stress, CORT levels were lower at 28 DPI compared with 7 DPI, and lower at 54 DPI compared with all other time-points post-injury. **(C)** Following 90 min of stress, CORT levels were lower at 14 DPI and 28 DPI compared with 7 DPI, and lower and 54 DPI compared with all other time-points. (+ denotes difference compared with 7 DPI, γ denotes difference compared with 14 DPI, ^ denotes difference compared with 28 DPI). All data are presented as mean ± SEM, *p* < 0.05. CORT, corticosterone; DPI, days post-injury; Mild TBI, mild TBI injury; Mod TBI, moderate TBI injury; SEM, standard error of the mean; TBI, traumatic brain injury.

We analyzed CORT levels measured at 30, 60, and 90 min post-restraint stress initiation to investigate if the repeated restraint stress paradigm resulted in habituation of the HPA axis as a function of time post-injury. There was a significant effect of time post-injury on CORT levels in response to stress where CORT levels lowered over DPI among all treatments (Fig. 5). An effect of stress but not injury was observed at 30 min post-stressor initiation. After accounting for the within-subject correlation between repeated measures via random effects ($\chi_{1}^{2}=11.05$, *p =* 0.0009), support existed for CORT levels decreasing over DPI (F[3,78] = 8.809, *p* < 0.0001; Fig. 5A). CORT levels measured at 30 min post-stressor initiation were on average 41.33 ng/mL lower at 28 DPI compared with 7 DPI, on average 51.27 ng/mL lower at 54 DPI compared with 7 DPI, and 40.4 ng/mL lower compared with 14 DPI. An injury effect on CORT at 30 min post-stressor initiation was not supported (F[2,27] = 0.990, *p =* 0.385; Fig. 5A), nor was an interaction between injury and time at the 30 min time-point (F[6,78] = 0.430, *p =* 0.857; Fig. 5A). At 60 min post-stressor initiation, CORT should be approaching the apex of increase. After accounting for the within-subject correlation between repeated measures via random effects ($\chi_{1}^{2}=20.55$, *p*<0.0001), our data indicated the CORT levels measured at this apex decreased over DPI (F[3,78] = 17.61, *p* < 0.0001; Fig. 5B).

CORT levels measured at the 60 min time-point were on average 40.48 ng/mL lower at 28 DPI compared with 7 DPI and on average 86.88 ng/mL lower at 54 DPI compared with all other time-points. This time effect was independent of an injury effect (F[2,27] = 0.554, *p =* 0.581; Fig. 5B). There was also no support for an interaction between injury and time at 60 min post-stressor initiation (F[6,78] = 2.164, *p =* 0.055; Fig. 5B). After accounting for the within-subject correlation between repeated measures via random effects ($\chi_{1}^{2}=7.195$, *p* = 0.007), CORT levels measured at 90 min post-stressor initiation also decreased as a function of DPI (F[3,78] = 19.62, *p* < 0.0001; Fig. 5C). CORT levels were on average 55.52 ng/mL lower at 14 DPI, 86.19 ng/mL lower at 28 DPI compared with 7 DPI, and 93.24 ng/mL lower at 54 DPI compared with all other time-points. An injury effect on CORT levels measured at the 90 min time-point was not supported (F[2,27] = 0.615, *p =* 0.548; Fig. 5C). There was also no interaction between injury and time at 90 min post-stressor initiation(F[6,78] = 0.769, *p =* 0.597; Fig. 5C). Outliers not included in analyses: 30 min time-point sham 54 DPI, *n =* 1; Mild TBI 28 DPI, *n =* 1; Mod TBI 28 DPI, *n =* 1; 60 min time-point Mild TBI 7 DPI, *n =* 1; Mild 54 DPI, *n =* 1; Mod TBI 14 DPI, *n =* 1.

### DEX suppressed stress-induced CORT

**FIG. 6.**
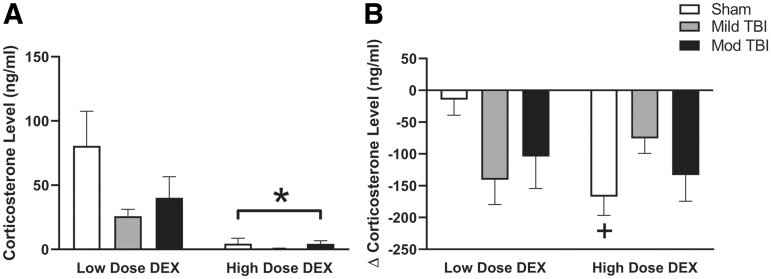
DEX suppressed stress-induced CORT. **(A)** Levels of CORT were tested 2 h following DEX injections (high dose, 0.1 mg/kg; low dose, 0.01 mg/kg) at 56 DPI. DEX suppressed CORT levels similarly in both the brain-injured rats and uninjured shams under stressed conditions. High-dose DEX suppressed CORT levels more than low-dose DEX, independent of TBI. **(B)** CORT levels following the administration of DEX at 56 DPI were compared with pre-stress CORT (0 min) collected at 54 DPI. Regardless of TBI, rats had lower CORT following DEX as indicated by a negative change in CORT levels. There was an interaction between injury and dose. The change in CORT was greater in high-dose shams compared with low shams. (* denotes significance compared with low-dose DEX, + denotes significance compared with low-dose DEX shams). All data are presented as mean ± SEM, *p* < 0.05. CORT, corticosterone; DEX; dexamethasone; DPI, days post-injury; Mild TBI, mild TBI injury; Mod TBI, moderate TBI injury; SEM, standard error of the mean; TBI, traumatic brain injury.

Following DEX treatment and restraint stress (90 min post-stressor initiation), there was a dose-dependent change in CORT (F[1,21] = 15.43, *p =* 0.0008; Fig. 6A). All groups administered high-dose DEX had on average 77.6 ng/mL lower CORT compared with low-dose shams. An overall injury effect on CORT was not supported (F[2,21] = 2.122, *p =* 0.145; Fig. 6A), nor was an interaction between injury and dose (F[2,21] = 2.122, *p =* 0.145). We further analyzed DEX-induced changes to CORT by calculating the change in CORT at 90 min post-stressor initiation following DEX at 56 DPI from the CORT measured 90 min post-stressor initiation at 54 DPI. Across all groups, DEX led to lower restraint-induced CORT release. This change was independent of an injury effect or dose-effect (Injury: F[2,21] = 0.275, *p =* 0.763; Dose: F[1,21] = 1.684, *p =* 0.209; Fig. 6B). However, support existed for a significant interaction between injury and dose (F[2,21] = 4.332, *p =* 0.027; Fig. 6B), and a significant difference in the change of CORT measured between low-dose and high-dose shams. Outliers not included in analyses: sham High Dose DEX, *n =* 1; Mild TBI High Dose DEX, *n =* 1; Mod TBI Low Dose DEX, *n =* 1.

## Discussion

We found that intermittent repeated administration of restraint stress, with 1–4 week rest periods, led to continual habituation of the HPA axis response. This is in line with previous studies where daily repeated exposure to homotypic stressors led to habituation of the HPA axis,^44^ and in opposition to studies that indicated reporting resting periods would diminish the habituation response.^43^

In this case, habituation refers to the progressive diminution of the magnitude of CORT release on repeated exposure to our restraint paradigm. Rats subjected to TBI and control shams habituated to repeated restraint stress, thereby supporting that repeated exposure to homotypic stressors can lead to habituation of the HPA axis. The mechanisms behind habituation are unclear but likely involve changes to glucocorticoid receptors (GR) and mineralocorticoid receptors (MR) in brain regions that regulate CORT release. Habituation to stressors is thought to be primarily mediated by MR,^45^ which are highly expressed in the hippocampus and prefrontal cortex, indicating a role for regions outside the direct HPA axis in mediating habituation.^46^ Also, treatment with MR antagonists has been associated with prevention of habituation to repeated restraint.^45^ At subacute time-points, GR and MR gene expression is similar between sham and brain-injured rats and mice,^47–49^ yet these levels have not been assessed longitudinally. Longitudinal impact of TBI on MR and GR expression in brain regions responsible for modulating the HPA axis warrants further investigation.

We evaluated resting CORT levels over time, independent of TBI. Our data indicated that resting CORT levels measured at the start of restraint stress were lower at 7, 14, 28, and 54 DPI in all rats compared with pre-surgery baseline levels. In this study, pre-surgery blood was collected between 04:00 and 07:00, which is prior to and immediately following light onset, respectively. Resting blood was drawn at the initiation of restraint stress between 07:30 and 09:00. Although blood collection times differed between pre-surgery baseline and post-injury time-points, this likely did not contribute to the chronic low levels of post-injury CORT measured in this study. A diurnal rhythm in plasma concentrations of CORT is well documented and conserved among species.^50–52^ The circadian nadir is reported just prior to light onset, followed by an increasing phase starting 4 to 6 h after the initiation of the light period, with the circadian peak occurring at the end of the light period and initiation of the dark period.^50,53,54^ Our pre-surgery baseline blood was collected at the circadian nadir of endogenous CORT and all post-injury collection times were within the first 6 h of the light period, approximately 6 h prior to the circadian peak of endogenous CORT production.

Our results provide considerable evidence that plasma CORT levels are elevated at both 6 and 24 h after diffuse TBI. This acute increase occurred following both mild and moderate TBI. Previous studies report that TBI elevates plasma CORT in the immediate hours following injury,^55^ which can potentially exacerbate neuronal and axonal injury.^56^ In this model, resting CORT levels were decreased at 54 DPI in the absence of repeated stressor exposure,^12^ coincidently similar to levels reported after repeated restraint stress in sham and injured rats in the present experiment. The etiology of affective symptoms and the influence of exposure to repeated stressors after TBI are poorly understood, partially due to the complex circuitry that mediates the responses.

Recent reports have evaluated the pathophysiology of modulatory brain regions of the HPA axis after TBI. In the paraventricular nucleus (PVN), no neuropathology was evident over 28 DPI, and glial fibrillary acidic protein (GFAP) was unchanged; however, microglia were activated at 7 DPI and there was a change in PVN neuron complexity.^12,49^ In the amygdala, TBI does not induce neuropathology over a 28-day time course; however, time-dependent and nuclei-dependent changes have been measured for microglial activation, astrocyte activation, neuron complexity, and glutamate neurotransmission.^57,58^ In areas of no pathology, changes in glial activation, neuron morphology, and glutamate neurotransmission are likely modulated by indirect processes that are poorly understood, but these changes overlap with reports in studies investigating the stress response, indicating similar mechanisms for disease progression.^12,49,57,58^ Common mechanisms between TBI and the stress response are translationally relevant for the validation of animal models to investigate the effectiveness of therapeutic targets.

As expected, restraint stress following the administration of a high dose of DEX resulted in lower CORT levels in both brain-injured and uninjured shams, without differences among groups. These data are in line with our previous work that indicated that high-dose DEX lowered CORT and brain-injured rats had an intact negative feedback system.^12^ In similar studies, DEX has been shown to suppress plasma CORT in rats following lateral FPI^59^ and controlled cortical impact.^40^ Here, we also tested a low dose of DEX because a high dose may saturate the system, subsequently occluding differences between brain-injured rats and shams. At 90 min post-restraint stress initiation, low-dose DEX suppressed CORT production in rats subjected to mild or moderate TBI, but not in uninjured shams when compared with CORT levels at 90 min post-restraint stress measured at 54 DPI. These data indicate diffuse TBI may alter the negative feedback of the HPA axis and further studies are needed to investigate this significant injury × dose interaction.

One limitation of our study was blood collection post-restraint stress. Our study design did not include blood draws after the rat was removed from the restrainer so it was not possible to discern if TBI impacted the time it took for restraint-induced CORT levels to return to baseline. Future studies that include longer collection times are needed to assess when the HPA axis resets, and how long it takes for restraint-induced CORT levels to return to baseline after a diffuse TBI. Another limitation of our study is that control sham rats were exposed to isoflurane, thus, the basal CORT and the reactivity of the HPA axis must be interpreted in the context of prior exposure to anesthesia. It is documented that brief exposure to an isoflurane anesthetic did not elevate ACTH or CORT levels in comparison with control, nor was it found to elevate circulating levels of adrenaline or noradrenaline in the plasma.^60–63^ In contrast, exposure to anesthesia over long durations in humans increased plasma cortisol levels measured acutely after inhalation.^64^ Further studies are needed to determine the long-term impact of isoflurane on the HPA axis regulation.

We only evaluated male rats, but human females tend to have elevated anxiety and psychiatric disorders following TBI, possibly due to biological sex differences in HPA axis function that include enhanced resting and stressor-induced activation.^65–67^ Sex has largely been ignored in pre-clinical TBI research, but evidence suggests sexual dimorphism exists, in part, due to endocrine-related differences, such as sex hormones.^47–49,68–71^ There is an active call to action to include females in pre-clinical TBI research to better inform clinical trials and effective treatments^70^; thus, our ongoing and future studies will extend and expand upon our current scope to include female rats.

Data presented in this article were collected from young adult rats, whereas both juvenile and aged rats may have a different CORT response. Hypopituitarism is a long-term complication of TBI in pediatric patients,^72^ and pituitary deficiencies have been reported following moderate-severe and mild injuries.^72–74^ We recently demonstrated that pediatric patients with a TBI have 3.22 times the risk of a subsequent central endocrinopathy compared with the general pediatric population.^74^ Aging may also represent a critical period of vulnerability, given senescence is a risk factor for the development of post-traumatic hypopituitarism.^75^ Future studies should include juvenile and aged rats.

The results from our study demonstrate the importance of using mixed-effects models to analyze data from repeated measures on the same subjects. Nearly all of our chi-square tests that compared the fit of models with and without random effects for individual rat supported the models that included random effects. This indicated that repeated measures for individual rats were correlated across time, and that non-negligible differences existed between rats within the same groups. Failure to account for such within-subject collinearity and within-group variation, respectively, could bias results and lead to flawed conclusions.

## Conclusion

Our results indicate that in the acute period following TBI, CORT is elevated compared with sham animals. However, in the weeks and months that follow TBI, CORT responses to stressors become indistinguishable from sham animals, indicating that intermittent restraint stress causes habituation, even when resting periods last 4 weeks. Future studies will continue to investigate changes to the brain regions that control the HPA axis following TBI. Understanding the acute rise in CORT following TBI may provide insight into the molecular underpinnings to secondary injuries and elucidate novel treatment strategies.

## Supplementary Material

Supplemental data

## Abbreviations Used

ANOVA: analysis of variance
ARRIVE: Animal Research: Reporting of In Vivo Experiments
CORT: corticosterone
DEX: dexamethasone
DPI: days post-injury
EDTA: ethylenediaminetetraacetic acid
ELISA: enzyme-linked immunosorbent assay
GR: glucocorticoid receptors
HPA: hypothalamic-pituitary-adrenal
mFPI: midline fluid percussion injury
Mild TBI: mild TBI injury
Mod TBI: moderate TBI injury
MR: mineralocorticoid receptors
NIH: National Institutes of Health
PVN: paraventricular nucleus
SEM: standard error of the mean
TBI: traumatic brain injury
