# Supplementary material for: Mild and Moderate Traumatic Brain Injury and Repeated Stress Affect Corticosterone in the Rat
Source: Neurotrauma Rep. 2020 Oct 21;1(1):113–24. doi: 10.1089/neur.2020.0019 (PMC8240883; doi:10.1089/neur.2020.0019)
Supplement: Supplemental data [file Supp_FigS1.pdf]

Supplementary Data

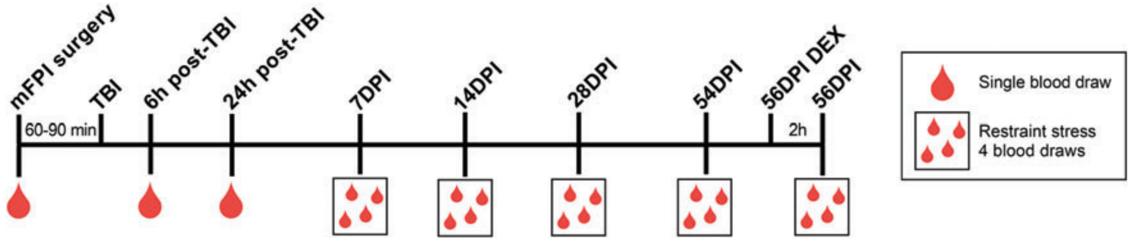

SUPPLEMENTARY FIG. S1. Study design. Young adult male rats were pair-housed and acclimated to the vivarium. A baseline blood draw was collected at the start of surgery followed by a 60–90 min recovery period. Rats were subjected to a diffuse traumatic brain injury (TBI) via a midline fluid percussion injury (mFPI) or sham surgery. A single blood draw was collected from all rats at 6 and 24 h post-injury. At 7, 14, 28, and 54 days post-injury (DPI) rats underwent restraint stress and blood collection. Rats were placed tightly into a restrainer and the first blood draw was immediately acquired (0 min). Rats remained in the restrainer for a total of 90 min (30 min of tight restraint, followed by 60 min of loosened restraint) and blood was collected at 30, 60, and 90 min post-restraint stress initiation. At 56 DPI, rats were randomly assigned to a low or high dose of dexamethasone (DEX) administered subcutaneously. Two hours post-injection the restraint stress paradigm was repeated, and blood was collected.
